# Fetal Cardiovascular Profile Score (CVPs) in Fetal Anemia, Using Fetal Hemoglobin Bart’s Disease at Mid-Pregnancy as a Study Model

**DOI:** 10.3390/diagnostics15182303

**Published:** 2025-09-11

**Authors:** Panisa Hantrakun, Kasemsri Srisupundit, Theera Tongsong

**Affiliations:** 1 Department of Obstetrics and Gynecology, Faculty of Medicine, Chiang Mai University, Chiang Mai 50200, Thailand; 2 Fetal Center, Faculty of Medicine, Chiang Mai University, Chiang Mai 50200, Thailand

**Keywords:** anemia, fetus, hemoglobin Bart’s disease, cardiovascular profile score (CVPs)

## Abstract

**Objectives:** To evaluate the diagnostic performance of CVPs in predicting fetal Hb Bart’s disease among pregnancies at risk and to study hemodynamic changes based on CVP components in response to fetal anemia. **Methods:** The database was assessed to retrieve the ultrasound records of fetuses at risk of Hb Bart’s disease at 17–22 weeks and the relevant files including complete video sets of fetal echocardiography. The five components of CVPs of each case were blindly assigned. The definitive diagnosis of fetal Hb Bart’s disease was based on cordocentesis or neonatal blood analysis. **Results:** Among 378 pregnancies at risk that were recruited into the study, there were 76 (20.1%) affected fetuses and 302 (79.9%) unaffected fetuses. Using a cut-off score of <9, CVPs had a sensitivity of 92.1% and specificity of 97.4% in predicting affected fetuses. However, the effectiveness was not much superior to cardio-thoracic area ratio (CTAR) alone (area under curve; AUC: 0.983 vs. 0.954). Of all parameters, CTAR provided the best diagnostic performance. The combination of CTAR and assessment of hydropic sign provided the best diagnostic values, comparable with full CVPs (AUC 0.982 vs. 0.983). The affected fetuses cope well with anemia by physically increasing in cardiac size and functionally increasing in Tei index with minimally reduced shortening fraction, without compromising arterial and venous Doppler indices. **Conclusions:** CVPs are highly effective in predicting affected fetuses among pregnancies at risk of fetal Hb Bart’s disease. Nevertheless, only two components (CTAR and hydropic sign) are adequate to yield the best diagnostic performance.

## 1. Introduction

Hemoglobin (Hb) Bart’s disease, or homozygous alpha0-thalassemia disease, is the most severe form of thalassemia disease and is almost always fatal in utero or shortly after birth. This form of the disease is the most commonly inherited disorder of hemoglobin synthesis in Southeast Asian countries [1,2,3], especially in Thailand [4,5]. The disease is caused by deletion of all alpha-globin genes, leading to non-production of alpha-globin chains. Hb Bart’s consists of four gamma globin chains, which have extremely high affinity to oxygen, resulting in tissue hypoxia, and are rapidly destroyed by the reticuloendothelial system [1]. These fetuses suffer from severe anemia, leading to a hemodynamic compensatory process, and then may progress to cardiomyopathy and fetal cardiac failure [6].

According to recent studies, sonographic examination is an effective method to identify fetuses at risk of hemoglobin Bart’s disease to avoid unnecessary invasive prenatal diagnosis procedures [7,8,9,10]. The cardiovascular profile (CVP) score, which was first reported by Huhta [11], combines five cardiovascular parameters, which are fetal hydrops, heart size, cardiac function, arterial Doppler, and venous Doppler of the umbilical vein (UV) and ductus venosus (DV), and assigns two points for each. A normal CVP score is 10, and a score of 5 or less predicted perinatal mortality [12,13]. Several studies have demonstrated that CVP score can be used as a predictor of severe fetal morbidity and mortality among various disorders such as congenital heart defects [14,15], fetal growth restriction [13], and fetal hydrops [12]. At present, there was no report about using the CVP score in evaluating cardiovascular changes in fetal anemia, particularly Hb Bart’s disease [16]. Theoretically, the CVP score may be useful in predicting fetal Hb Bart’s disease since the affected fetuses are expected to show some cardiovascular changes, which are components in the CVP score. Accordingly, we hypothesize that reduced CVPs may be helpful in differentiating the affected fetuses from the non-affected ones. The objectives of this study are primarily to evaluate the diagnostic performance of CVPs in predicting fetal Hb Bart’s disease among pregnancies at risk and to study hemodynamic changes based on CVP components in response to fetal anemia using fetal Hb Bart’s disease as a study model.

## 2. Patients and Methods

This study was the diagnostic test. After being ethically approved by the Institute Review Board (Study Code: OBG-2566-0205, 20 June 2023), the databases of maternal–fetal medicine unit of Maharaj Nakorn Chiang Mai Hospital, Faculty of Medicine, Chiang Mai University from January 2010 to December 2022, were reviewed and recruited into the study. Inclusion criteria were singleton pregnant women, gestational age 16–22 weeks, couples at risk for fetal hemoglobin Bart’s disease, and whose who either received a prenatal diagnosis procedure or a serial ultrasonographic examination for pre-hydropic signs. The gold standard for diagnosis of fetal hemoglobin Bart’s disease was either cordocentesis for hemoglobin typing, using high-performance liquid chromatography, or neonatal blood analysis. Exclusion criteria were fetuses with major anomalies or chromosomal abnormalities (except pre-hydropic signs of fetal hemoglobin Bart’s disease), fetuses with anemia or hydrops from non-Bart’s disease and unknown diagnosis, and incomplete data. The database of ultrasound clips was in DICOM format, and images were recruited into the study for subsequent offline analysis. The video clips and images were consecutively selected and assigned by the senior author (TT) to the first author (PH), who was blinded to the diagnosis, for scoring.

All sonographers were Maternal–Fetal Medicine (MFM) fellows or MFM staff, using a Voluson E10 machine (GE Healthcare Ultrasound, Milwaukee, WI, USA), equipped with transabdominal 2- to 4-MHz curvilinear transducers as well as a 3D convex transabdominal 3.5-MHz transducer. The maximal Doppler intensity was maintained at less than 100 milliwatts/cm^2^ spatial peak temporal average. The high-frequency filter was set at 125 Hz to remove signals from slow-moving tissues during cardiac examination and set at 50 Hz during arterial and venous Doppler examinations.

The baseline characteristics of the study population were recorded, and the database of ultrasound clips and images was evaluated for CVP score as shown in Table 1.

**The Statistical Analysis:** All statistical procedures were performed using the statistical package for the social sciences (SPSS) software version 26.0 (IBM Corp. Released 2019). IBM SPSS Statistics for Windows, Version 26.0: IBM Corp.). The performance of the cardiovascular profile (CVP) score in the diagnosis of the affected fetuses was calculated and presented as sensitivity, specificity, positive predictive value, and negative predictive value, as well as a 95% confidence interval (CI). A *p*-value of less than 0.05 was considered statistically significant.

Comparisons of baseline characteristics and cardiac parameters between affected and unaffected groups were performed using Student’s T-test or the Mann–Whitney U-test for continuous data, according to the normality of distribution, and chi square for categorical data. The area under the receiver operating characteristic curve (AUC) was used to assess model performance. Differences in AUCs among the predictors were evaluated using DeLong’s method (paired-sample design). As a diagnostic test study, the sample size was calculated based on an estimated sensitivity of 95%, as reported in previous studies, with a 95% confidence interval and an allowable margin of error of 0.05 (5%). Given a disease prevalence of 25% for this autosomal recessive condition, the required sample size was determined to be at least 292 fetuses at risk.

## 3. Results

During the study period, a total of 428 pregnant women were recruited into the study. Among them, 378 pregnancies had complete data for analysis, consisting of 76 (20.1%) affected fetuses and 302 (79.9%) unaffected fetuses. The baseline characteristics of the two groups were not significantly different, except that hemoglobin levels were significantly lower in the affected group, as presented in Table 2. The mean (±SD) gestational age at the time of recruitment was 18.9 ± 1.5 weeks.

The median CVP score was significantly lower in the affected group compared to unaffected fetuses, which were 8 and 10, respectively. In comparisons of cardiac parameters between the two groups, cardiac size, either cardiac to thoracic diameter or area ratio, was significantly higher in the affected fetuses, as presented in Table 3. The affected fetuses had a significant decrease in global sphericity index (GSI), which reflects cardiac remodeling. Functionally, the Tei index on both sides was significantly increased in the affected fetuses. Moreover, the shortening fraction of the right ventricle was significantly lower in affected fetuses.

The sensitivity and specificity of the CVP score in predicting the affected fetuses varied, depending on the defined cut-off, as presented in Table 4. The cut-off value of 9 gives the highest sensitivity in the detection of fetal Hb Bart’s disease, which was 92.1%, and a specificity of 97.4%, a positive likelihood ratio of 12.3, a negative predictive value of 0.03, and a Youden index of 0.895.

The performance of each component of the cardiovascular profile (CVP) score in predicting the fetuses affected by Hb Bart’s disease are presented in Table 5. Of the five parameters, cardiomegaly gave the highest accuracy with 89.5% sensitivity and 98.0% specificity, followed by fetal hydrops, cardiac function, and Doppler study, respectively. Note that the combination of cardiomegaly and fetal hydrops can improve the sensitivity of diagnosis from 89.5 to 92.1%, without compromising the specificity.

**Figure 1:**
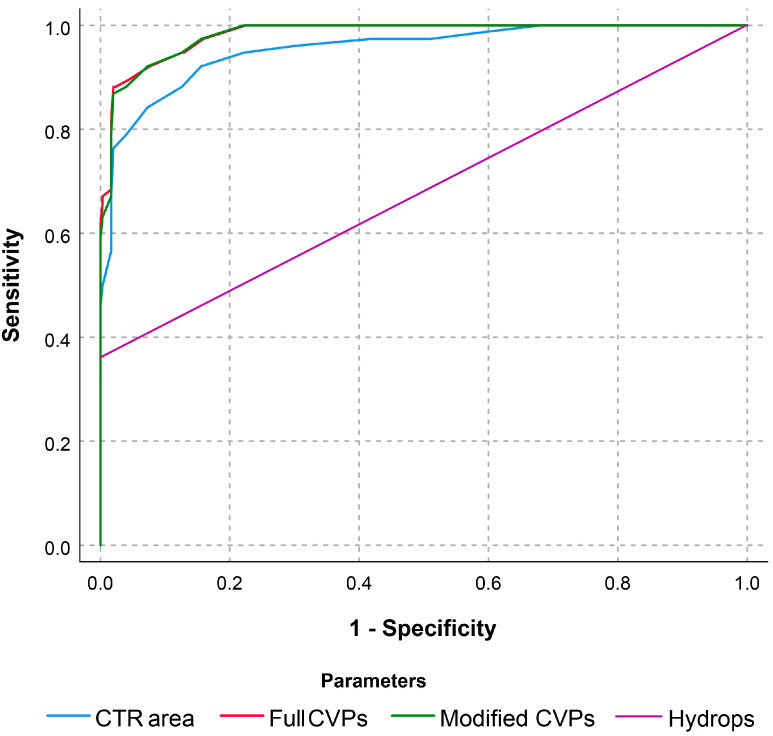
A comparison of receiver operating characteristic curves between the full CVP score, modified CVP score, CTAR, and hydrops in predicting Hb Bart’s disease at mid-pregnancy (area under curve 0.983 [95% CI: 0.972–0.994], 0.982 [95% CI: 0.971–0.993], 0.954 [95% CI: 0.927–0.980] and 0.671 [95% CI: 0.593–0.749] for full CVP score, modified CVP, CTAR, and hydrops, respectively).

In comparisons of diagnostic performances of CTAR, modified CVPs (defined as a combination of CTAR and hydrops fetalis), and full CVPs, the ROC curves are constructed, as presented in Figure 1, based on CTAR, and probabilities of affected fetuses are calculated from the logistic regression models derived from various components of modified CVPs and full CVPs. The ROC curves indicate that the performance of modified CVPs and full CVPs was minimally, but significantly, superior to that of cardiac size alone (AUCs: 0.982 vs. 0.954, *p*-value: 0.020; and 0.983 vs. 0.954, *p*-value: 0.017, respectively). Importantly, the AUCs of modified CVPs and full CVPs were not significantly different (*p*-value: 0.361), indicating that adding other components of CVPs (cardiac function, umbilical artery Doppler, and venous Doppler) to the cardiac size does not add to the overall accuracy.

Subgroup analysis was performed to compare the prevalence of abnormality of other CVP components (score 0 or 1), which are cardiac function, umbilical artery Doppler and venous Doppler, and mean CTAR between the affected fetuses with and without hydropic signs, as presented in Table 6. Note that fetal cardiac size and cardiac function, as well as vascular Doppler studies, were not significantly different between the affected fetuses with hydropic signs and those without hydropic signs.

## 4. Discussion

**New Insights:** Insights gained from this study are as follows: (1) CVP score is highly effective in predicting affected fetuses among pregnancies at risk of fetal Hb Bart’s disease. However, the effectiveness was not much superior to CTAR alone. (2) Of the five parameters of the CVP score, CTAR provided the best performance in predicting the affected fetuses. (3) The combination of CTAR and assessment of hydropic sign provided the best diagnostic values. (4) A full CVP score of the five parameters was not necessary in the evaluation of fetal anemia secondary to Hb Bart’s disease at mid-pregnancy. (5) The fetuses cope well with anemia by physically increasing in cardiac size and functionally increasing in Tei index and possibly reducing shortening fraction (but all were within normal limit) without compromising UA blood flow and venous Doppler indices. In other words, in response to anemia, the fetal heart works harder but works well, likely caused by a decrease in afterload secondary to peripheral anemic hypoxia. (6) In fetal anemia, hydrops fetalis is probably a consequence of hypervolemia, rather than a sign of high-output heart failure.

This study provides information that the cutoff point of the CVP score for prediction of fetal Hb Bart’s disease among fetuses at risk at mid-pregnancy was a score of 9 (CVP < 9). In contrast to previous studies about CVP score, the cutoff point of CVP score < 5 is used as a predictor of severe fetal morbidity and mortality among various disorders such as congenital heart defects, fetal growth restriction, and fetal hydrops [12,13]. Nevertheless, the purpose of CVPs in our study is different; they are not used to predict fetal morbidity and mortality but to differentiate the affected from the non-affected fetuses who are at risk of anemia.

**Interesting Findings of *Cardiac Function:*** Despite hypervolemia and increased cardiac output to increase tissue perfusion, cardiac function seemed to increase but was within normal limits, though shortening fraction slightly reduced and Tei index increased. Based on UA and venous Doppler, the heart was not associated with pressure load despite volume load. This is likely due to a decrease in afterload secondary to peripheral vasodilation in response to anemic hypoxia. Note that trivial TR was commonly seen in the affected fetuses, probably representing incomplete closure of the tricuspid valve because of enlarged AV annulus secondary to cardiomegaly, rather than representing poor cardiac function. Though increased cardiac function was more commonly seen in the affected fetuses, omitting this parameter from the CVPs did not seem to compromise the predictive value because these fetuses had cardiomegaly in nearly all cases of abnormal function.

***Hydrops Fetalis:*** In fetal anemia, hydrops fetalis can develop despite no cardiac dysfunction or no heart failure, different from a fetus with FGR or a recipient twin of TTTS in which hydrops develops only when heart failure occurs. Accordingly, hydrops fetalis in the affected fetuses was not associated with high-output heart failure but rather caused by fluid shift due to volume load, reduced colloid oncotic pressure, and hypoxia-induced capillary damage. Our findings suggest that hydrops fetalis in fetuses with anemia is expected to have a better prognosis than that in fetal cardiac disease or high afterload conditions like FGR.

***UA End-Diastolic Flow:*** The pulsatility index of the UA was not significantly different between the affected cases and controls, indicating a decrease in placental resistance or afterload to maintain normal end-diastolic flow, despite volume load in fetal anemia. Accordingly, our observation was consistent with the findings in previous studies, which supported that a decrease in afterload is a primary mechanism of compensation in anemia [18,19,20]. A decrease in afterload in fetal anemia might be caused by a reduction of serum viscosity [21], peripheral vasodilation [22], and endothelial dysfunction [23]. Nevertheless, note that the UA pulsatility index in the affected group tended to be higher. This might imply that the compensatory mechanism tends to gradually become exhaustive, though not yet abnormal, and possibly lead to high-output heart failure eventually.

***Venous Pulsations:*** UV pulsations in our affected fetuses were unlikely to represent increased preload, reflexive of cardiac decompensation, because all of them have normal or high a-waves in the ductus venosus, different from UV pulsations commonly seen in fetuses with increased afterload like FGR as well as increased preload because of high venous pressure (absent or reversed a-waves in DV). We hypothesize that the contradictory findings of such venous Doppler waveforms might be associated with hypervolemia causing dilatation of the DV, which is a sphincter-like structure, permitting atrial pulsations propagating through the DV to the UV. Accordingly, UV pulsations together with normal or increased DV a-waves should not be considered as a sign of cardiac dysfunction or increased preload. Additionally, in the case of UPI, UV pulsations are typically present together with AEDV/REDV in the UA, different from our findings, which showed UV pulsations despite normal or even high diastolic flow in the UA.

**Proposed Pathophysiology:** According to evidence mentioned above, we hypothesize that fetal anemia causes a decrease in tissue oxygen perfusion, leading to activation of several adaptive mechanisms to improve tissue oxygenation, primarily increasing cardiac output by increasing cardiac size and cardiac function, together with a decrease in afterload by increasing peripheral vasodilation, as indicated by maintaining UA end-diastolic flow despite volume load. Accordingly, the response to fetal anemia is characterized by an increase in volume load with less pressure load and minimal cardiac dysfunction without increasing preload pressure as indicated by normal venous Doppler waveforms. Note that pressure load in the fetal heart may probably be mitigated by decreased afterload. Therefore, fetal heart failure is less likely to develop despite longstanding anemia and the presence of hydrops fetalis. Volume load in fetal circulation can cause fluid leakage to the extravascular compartment, resulting in hydrops fetalis without obvious cardiac failure.

**Clinical Implications:** This study showed that cardiac size is highly effective in predicting the affected fetuses, consistent with that in previous studies [7,9,24,25,26]. Nevertheless, this study adds that the combination of cardiac size and the presence of hydropic signs can improve the diagnostic performance. Thus, this study provides evidence that, in addition to usefulness in predicting perinatal morbidity and mortality, CVPs may probably be used as an attractive alternative for differentiating the anemic fetuses among pregnancies at risk. Accordingly, assessment of cardiac size and hydropic signs (modified CVPs) should be routinely performed among pregnancies at risk of fetal anemia, while full CVPs seem less helpful in routine practice. Assessment of cardiac size and detection of hydropic signs are relatively simple procedures that can be completed within 5–10 min and are readily feasible for general obstetricians, given that cardiac size evaluation is already an integral component of the routine mid-trimester anomaly ultrasound screening. In cases with abnormal findings, referral to MFM specialists or experienced sonographers may be warranted, as evaluations of cardiac function and venous Doppler studies are typically performed by these specialists. The good diagnostic performance of CVPs shown in this study might be hypothetically reproducible in fetuses at risk of anemia due to other causes. Nevertheless, clinical application in anemia due to other causes is yet to be further evaluated. Certainly, though our results could not perfectly be applied to anemia due to other causes, similar adaptations to anemia at various magnitudes may be expected. Perhaps, decisions on intrauterine treatment should not be based on Hb levels alone as seen in traditional practice, but rather cardiac function, and venous and arterial Doppler should also be incorporated. Likewise, hydrops fetalis in fetal anemia may not always be an ominous sign because of heart failure, as seen in cases of increased afterload, and may not necessarily be an indication of delivery for postnatal treatment.

**Research Implications:** This study demonstrated the effectiveness of CVPs in predicting the affected fetuses among fetuses at risk of Hb Bart’s disease and hemodynamic changes in response to fetal anemia. Nevertheless, our results should be externally validated by further studies in other populations. Additionally, fetal anemia secondary to other causes may have a different natural course of anemia, though similar adaptation can be expected. Future studies on the effectiveness of CVPs and hemodynamic adaptation to fetal anemia due to other common causes such as parvovirus B19, isoimmunization, and feto-maternal hemorrhage should be conducted. Additionally, studies on the incorporation of MCA-PSV, which is well accepted as a predictor of fetal anemia, and the CVP component should be conducted. The correlation between fetal hemoglobin levels and cardiovascular change should be studied, since guidelines of intrauterine transfusion, solely depending on Hb levels, are based on very limited data.

**Strengths and Limitations:** The strengths of this study are as follows: (1) The sample size is large enough to address the main objectives with high reliability of the conclusion; (2) high-homogeneity of the participants in terms of gestational age, racial factors, and the cause of fetal anemia; (3) the analyses cover the performance of the overall full CVP score and each parameter of the CVP score; and (4) hemodynamic changes in response to fetal anemia in this study are highly reliable because of not being confounded by therapeutic intervention. The limitations of this study include (1) The assessments of CVPs were performed by MFM specialists. The reproducibility when performed by general obstetricians needs to be evaluated. (2) This study did not use CVP score as a predictor of fetal morbidity or mortality, but we rather used CVP score to differentiate anemic fetuses from non-anemic fetuses. (3) Though this study demonstrates the effects of long-standing anemia, unfortunately, we could not follow the natural course of anemia among these fetuses until term or when high-output heart failure developed because all the affected fetuses were terminated shortly after diagnosis. (4) Although the physician assessed the CVP scores blinded to the diagnosis, the clips or images of the affected fetuses might have revealed sonographic clues suggestive of Hb Bart’s disease, which could have compromised the blinding. (5) Inter- and intra-observer variability were not evaluated.

## 5. Conclusions

In conclusion, this study demonstrates that a CVP score of 9 or less as a cut-off point can effectively predict the fetuses affected by Hb Bart’s disease among pregnancies at risk. Nevertheless, the effectiveness in prediction was not superior to cardiac size as a single parameter. Of the five parameters of a CVP score, CTAR is the most useful in the assessment to predict fetal Hb Bart’s disease among fetuses at risk at mid-pregnancy, followed by fetal hydrops, whereas the remaining parameters were very low in sensitivity for prediction. Adding the assessment of hydropic signs to cardiac size measurement could improve the sensitivity in prediction without compromising the specificity, with no need of extra effort in practice. Therefore, the combination of these two parameters of CVPs seems to be most attractive in the application of CVPs in the evaluation of fetal anemia. Nevertheless, reproducibility in clinical practice, especially in fetuses with anemia secondary to other causes, must be elucidated by further studies. This study also helps better understand the pathophysiology of fetal adaptation to anemia. The fetuses cope well with anemia by physically increasing in cardiac size and functionally increasing in Tei index and possibly reducing shortening fraction, without compromising UA blood flow and venous Doppler indices. In response to anemia, the fetal heart works harder but works well, likely caused by a decrease in afterload secondary to peripheral anemic hypoxia. In fetal anemia, hydrops fetalis is probably a consequence of hypervolemia, rather than a sign of high-output heart failure.

## Figures and Tables

**Table 1:** The cardiovascular profile (CVP) score [12,14,17].

Category	Score 2	Score 1	Score 0
Fetal hydrops	None	Ascites or pleural effusion or pericardial effusion	Skin edema
Heart size; cardiomegaly (cardio-thoracic area ratio: CTAR *)	>0.20 and <0.35	0.35–0.50	>0.50 or <0.20
Cardiac function	Normal TV and MV, RV/LV SF > 0.28, biphasic diastolic filling	Holosystolic TR or RV/LV SF < 0.28	Holosystolic MR or monophasic diastolic filling
Arterial umbilical Doppler	Positive EDV	UA AEDV	UA REDV
Venous Doppler UV and DV	Non-pulsatile UV and normal DV	Non-pulsatile UV and negative A-wave in DV	Pulsatile UV

**Note:** TV = tricuspid valve, MV = mitral valve, TR = tricuspid regurgitation, MR = mitral regurgitation, RV = right ventricle, LV = left ventricle, SF = shortening fraction, UA = umbilical artery, EDV = end-diastolic velocity, AEDV = absent end-diastolic velocity, REDV = reversed end-diastolic velocity, UV = umbilical vein, DV = ductus venosus. (* For CTAR measurement, both the heart and thorax were measured using the electronic ellipse method on the same still two-dimensional ultrasound image, obtained at end-diastole in the standard four-chamber view. First, an ellipse was placed to encompass the cardiac apex, the outer epicardial borders, and the upper edge of the atrial septum. The cardiac area was then automatically calculated using the software integrated into the ultrasound machine. A second ellipse was positioned to encircle the thoracic cavity, including the posterior edge of the vertebra, the outer rib borders, and the anterior chest wall, while excluding subdermal tissues. The thoracic area was then automatically calculated. Finally, the fetal cardiothoracic (CT) ratios were derived by dividing the cardiac circumference by the thoracic circumference and the cardiac area by the thoracic area, respectively.

**Table 2:** Baseline characteristics of study population.

Baseline Characteristics	Affected Fetuses (*n* = 76)	Unaffected Fetuses (*n* = 302)	*p*-Value
Maternal age (year)	27.9 ± 6.3	28.9 ± 5.9	0.180 ^#^
Gestational age at ultrasound examination (weeks)	18.8 ± 1.8	18.9 ± 1.4	0.426 ^#^
Biparietal diameter (cm)	4.17 ± 0.46	4.25 ± 0.38	0.157 ^#^
Parity (missing data in 32 cases)			0.523 ^##^
Nulliparous	39	142	
Parous	31	134	
Hemoglobin levels (g/dL)	5.8 ± 1.7	10.4 ± 1.4	<0.001 *

^#^ Student’s *t* test; ^##^ Chi-square test; * indicative of statistical significance.

**Table 3:** Comparisons of cardiac and Doppler parameters between affected and unaffected fetuses.

Cardiac Parameters	Affected Fetuses (*n* = 76)	Unaffected Fetuses (*n* = 302)	*p*-Value
CVP score: median (IQR)	8 (2)	10 (1)	<0.001 *
Cardiac–thoracic diameter ratio (CTR)	0.61 ± 0.07	0.49 ± 0.05	<0.001 ^#^ *
Cardiac–thoracic area ratio (CTAR)	0.40 ± 0.06	0.28 ± 0.04	<0.001 ^#^ *
Global sphericity index (GSI)	1.14 ± 0.27	1.26 ± 0.08	<0.001 ^#^ *
Left Tei index	0.56 ± 0.10	0.49 ± 0.09	<0.001 ^#^ *
Right Tei index	0.57 ± 0.12	0.51 ± 0.09	<0.001 ^#^ *
Left ventricular shortening fraction	40.02 ± 12.07	42.49 ± 12.76	0.129 ^#^
Right ventricular shortening fraction	35.91 ± 9.66	39.91 ± 9.06	0.001 ^#^ *
Umbilical artery pulsatility index median (IQR)	1.38 (0.44)	1.29 (0.27)	0.054 ^##^

^#^ Student’s *t* test; ^##^ Mann–Whiney-U test; * indicative of statistical significance.

**Table 4:** Sensitivity, specificity, positive predictive value (PPV), and negative predictive value (NPV) of the CVP score in different cutoff points.

The CVP Score: Cutoff Points	Affected (*n* = 76)	Unaffected (*n* = 302)	Sensitivity, % (95% CI)	Specificity, % (95% CI)	PPV, % (95% CI)	NPV, % (95% CI)
CVP ≤ 9	70	8	92.1 (86–98.2)	97.4 (95.5–99.2)	89.7 (83.0–96.5)	98.0 (94.9–100)
CVP ≤ 8	40	0	52.6 (41.4–63.9)	100 (100–100)	100 (100–100)	89.3 (79.8–98.9)
CVP ≤ 7	24	0	31.6 (21.1–42)	100 (100–100)	100 (100–100)	85.3 (71.1–99.5)
CVP ≤ 6	6	0	7.9 (1.8–14)	100 (100–100)	100 (100–100)	81.2 (49.9–100)

**Table 5:** Sensitivity, specificity, PPV, and NPV of various parameters of the CVP score in predicting Hb Bart’s disease at mid-pregnancy.

Abnormal Parameters of CVP Score (Score 0 or 1)	Affected (*n* = 76)	Unaffected (*n* = 302)	Sensitivity, % (95% CI)	Specificity, % (95% CI)	PPV, % (95% CI)	NPV, % (95% CI)
Fetal hydrops	26	0	34.2 (23.5–44.9)	100 (100–100)	100 (100–100)	85.8 (72.4–99.2)
Cardiomegaly	68	6	89.5 (82.6–96.4)	98.0 (96.4–99.6)	91.9 (85.7–98.1)	97.4 (93.7–100)
Cardiac function	18	2	23.7 (14.1–33.2)	99.3 (98.4–100)	90 (76.9–100)	83.8 (67.7–99.9)
Arterial umbilical Doppler	4	0	5.3 (0.2–10.3)	100 (100–100)	100 (100–100)	80.7 (42.1–100)
Venous Doppler UV and DV	4	0	5.3 (0.2–10.3)	100 (100–100)	100 (100–100)	80.7 (42.1–100)
Combined hydrops and cardiomegaly	70	6	92.1 (86.0–98.2)	98.0 (96.4–99.6)	92.1 (86.0–98.2)	98 (94.9–100)

**Table 6:** Comparisons of each CVP component between the affected fetuses with and without hydrops fetalis.

CVP Component	Fetal Hydrops		*p*-Value
	Presence (*n*: 26)	Absence (*n*: 50)	
Cardiac–thoracic area ratio (CTAR) (mean ±SD)	0.41 ± 0.07	0.40 ± 0.06	0.375 *
Abnormal cardiac function	8 (30.8%)	10 (20.0%)	0.295 **
Abnormal arterial umbilical Doppler	3 (11.5%)	1 (2.0%)	0.113 ^#^
Abnormal venous Doppler	2 (7.7%)	2 (4.0%)	0.603 ^#^

* Student’s *t* test; ** chi square test; ^#^ Fisher Exact test.

## Data Availability

The data that support the findings of this study are available from the corresponding author upon reasonable request.
